# Emphysematous cholecystitis in a young male without predisposing factors: A case report

**DOI:** 10.1097/MD.0000000000005367

**Published:** 2016-11-04

**Authors:** Ming-Yu Chen, Chen Lu, Yi-fan Wang, Xiu-Jun Cai

**Affiliations:** Department of General Surgery, Sir Run Run Shaw Hospital, College of Medicine, Zhejiang University, Hangzhou, Zhejiang Province, China.

**Keywords:** emphysematous cholecystitis, laparoscopic cholecystectomy, predisposing factors

## Abstract

This report describes the diagnosis and treatment for Emphysematous cholecystitis (EC) without predisposing factors, and reviews the current literature.

A 49-year-old male without predisposition presented to emergency department with a two-day history of sudden onset abdominal pain, hypertension and received empirical antibiotics with Imipenem/Cilastatin 0.5 g via intravenous route every 8 hours. Computed tomography (CT)-scan revealed that air encircling gallbladder is the most important and accurate evidence for EC diagnosis.

Laparoscopic cholecystectomy was performed, and no stone was seen in gallbladder.

The patient's temperature and pulses returned to normal following laparoscopic cholecystectomy. The festering bile culture report showed E.coli and pathological analysis of the resected gallbladder disclosed that necrosis and mild mucosal dysphasia. The patient fully recovered without complication at outpatient clinic visit three months later.

The EC is an acute infection of the gallbladder wall caused by gas-forming organisms, is a life-threatening cholecystitis with mortality rate as high as 25%. Therefore, the combination of laparoscopic cholecystectomy and antibiotics is recommended as soon as possible once the diagnosis of EC was a clean-cut.

## 1 Introduction

Emphysematous cholecystitis (EC) is one of the most life-threatening forms of acute cholecystitis, with low morbidity, averaging from 1% to 3%. However, the mortality rate is as high as 25%.^[1]^ Presentation of EC with metabolic disorders such as diabetes mellitus, and immunosuppressed and peripheral vascular disease is common,^[2]^ but diagnosis is rather difficult. Patients usually present with sudden-onset right upper quadrant pain in the abdomen, fever, vomiting, and jaundice. Hence, it is very challenging to distinguish EC from gallstone-related acute cholecystitis or acute obstructive suppurative cholangitis (AOSC) based on symptoms and signs. Imaging showing gas in gallbladder walls or lumen is the most important and accurate clinical characteristic for diagnosing of EC.^[3]^ There is no doubt that the postoperative pathological analysis of resected gallbladder is the gold standard, yet it cannot provide us any indications before the operation. There are few literatures reported (Table 1); therefore, we decided to share our experience in the diagnosis and treatment of EC.

**Table 1:**
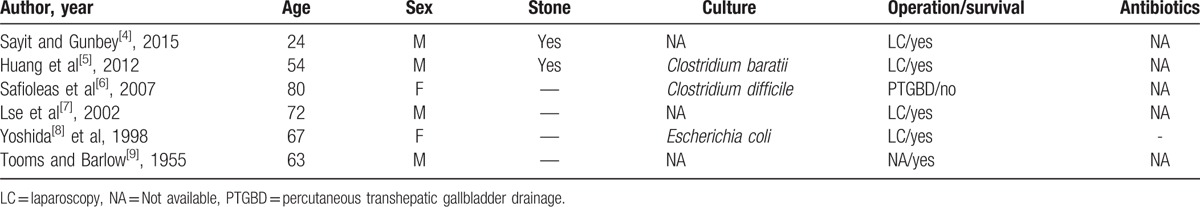
Review of case report of emphysematous cholecystitis without predisposing factors.

## 2 Case presentation

**Figure 1:**
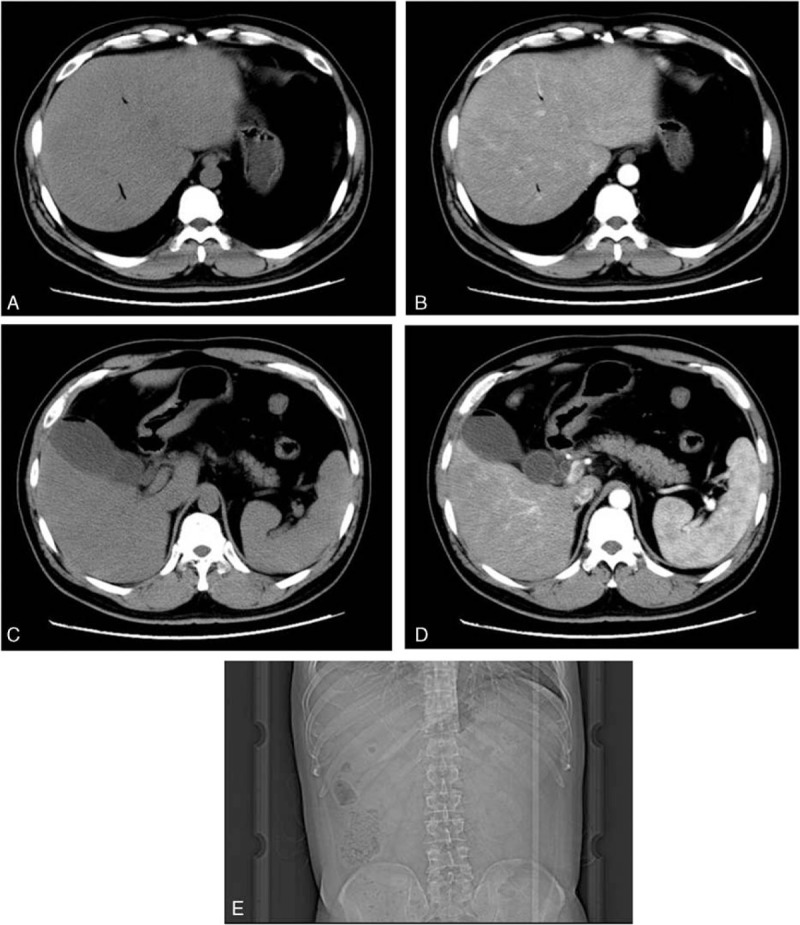
Computed tomography (CT)-scan (A-D) and plain-film radiography (E) showing air in the gallbladder wall or lumen.

**Figure 2:**
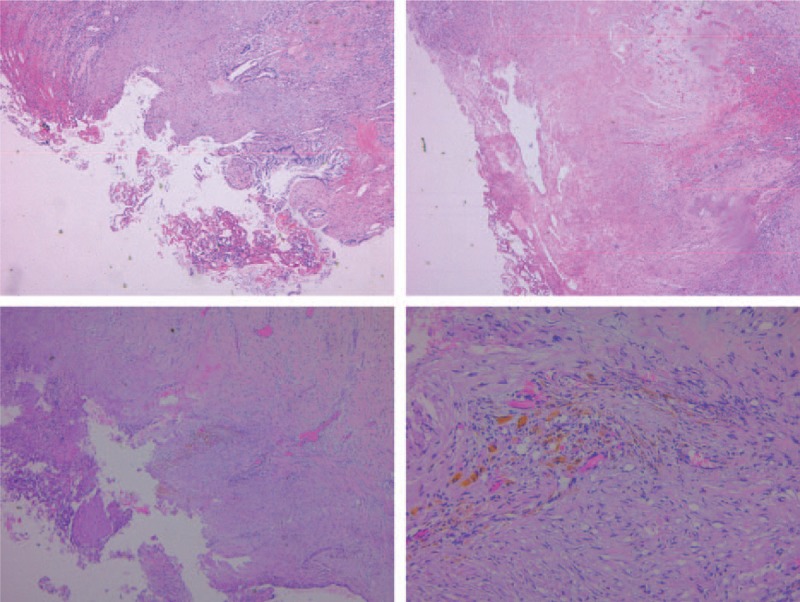
Pathologic analysis of the resected gallbladder.

A 49-year-old male presented to the emergency department at the Sir Run Run Shaw Hospital, China, complaining of a 2-day history of sudden-onset nonradiating abdominal pain in the right upper quadrant with Murphy sign, muscular defense of the upper abdomen, fever (38.5–39°C) without vomiting, or jaundice. The patient did not have a history of diabetes mellitus, immunosuppressed, peripheral vascular disease, or hepatic disease. Although his blood pressure was 150/85 mm Hg, pulse of 120 per minute, the respiratory rate was normal at 19 per minute. The result of a computed tomography (CT)-scan revealed air encircling the gallbladder and intrahepatic bile duct without gallstones (Fig. 1). The patient was then prescribed with empirical antibiotics imipenem/cilastatin 0.5 g by intravenous infusion every 8 hours. Emergency laparoscope was performed. Around 10 mL of gas and festering bile was collocated by needle to culture, determining the presence of gas-forming organisms causing acute cholecystitis. Laparoscopic cholecystectomy showed no gallstones. The resected gallbladder was delivered for pathological analysis. The patient's temperature and pulses returned to normal after laparoscopic cholecystectomy. The festering bile culture report was *Escherichia coli* and pathological analysis of the resected gallbladder showed that necrosis and mucosal dysplasia graded mild (Fig. 2). Three months at follow-up, the patient was fully functional without complication.

## 3 Discussion

Emphysematous cholecystitis is a rare variant of acute infection of the gallbladder wall caused by gas-forming organisms. Stoltz et al^[10]^ reported the first case of EC in 1990, and Garcia-Sancho et al^[1]^ described and reported its clinical features with mortality rates up to 25% and the morbidity rates up to 50%, which are much higher in complicated patients compared with uncomplicated patients (1%–3% and 15%, respectively).^[1,11,12]^ There are various predisposing factors including diabetes mellitus, immunosuppression, peripheral vascular disease,^[2]^ abdominal surgery, and trauma.^[4]^ EC is particularly common in older patients with diabetes mellitus because ischemia environments in diabetic patients reduce phagocytes’ mobility in the areas of infection and further reduce antimicrobial activity.^[13]^ Appropriate glycemic control can lower the likelihood of bacterial overgrowth and associated disease severity. In our case, the patient was 49 years old without diabetes, which made the diagnosis a bit more difficult and arduous.

There are some differences between EC and gallstone-related acute cholecystitis in its pathophysiology and epidemiology.^[14]^ The symptoms of EC are almost identical with those of acute cholecystitis, right upper quadrant pain, fever, vomiting, and jaundice, but EC begins as acute cholecystitis, then develops into ischemia and gangrene in the gallbladder wall with gas encircling the gallbladder due to gas-forming organisms, including *E coli, Clostridium welchii*, *Perfringens, Klebsiella*, and *Streptococci*.^[1,3,7,15,16]^ Hence, gas encircling the gallbladder or air within the gallbladder becomes the typical presentation of EC from the CT-scan.^[3,14]^ Of courses, ultrasonography (USG) and plain radiography can also be used for the diagnosis of EC. However, USG, which almost depends on the number of air pockets and on localization in the soft tissues, is an operator-dependent and less sensitive technique,^[12,17]^ whereas a gaseous halo around the gallbladder and gas-fluid level in the gallbladder can be shown more clearly by plain radiography and CT-scan. Moreover, CT-scan can detect pericholecystic edema and exclude other differential diagnosis.^[18]^ In a word, the imaging of CT-scan presenting gas encircling the gallbladder wall or lumen is the most important and accurate clinical characteristic for EC. CT-scan is the first choice for us to differentially diagnosing possibilities including AOSC, perforation, and acute pancreatitis.

In addition, laboratory examination consisting of C-reactive protein and liver function [aspartate aminotransferase (AST) and alanine aminotransferase (ALT)] cannot be neglected. The pus and blood culture also contribute to the diagnosis. Broad-spectrum antibiotics should be used to prevent worsening infection, and continued until blood and pus culture is reported. Then, according to aerobic culture report, we should switch to the antibiotics that bacteria are sensitive to.

Reviewing published literature, the broad-spectrum antibiotics and adequate surgical interventions should be taken into practice as soon as possible.^[3,18,19]^ The laparoscopic cholecystectomy is recommended as an effective and safe approach.^[15]^ If the doctor is unable to perform laparoscopic cholecystectomy, the gallbladder drainage can be considered.

## 4 Conclusions

Emphysematous cholecystitis is an unusual life-threatening variant of acute infection of the gallbladder wall caused by gas-forming organisms. The imaging of CT-scan presenting gas encircling gallbladder wall or lumen is the most important and accurate method for differential diagnosis. The combination of laparoscopic cholecystectomy and antibiotics is regarded as an effective and safe approach on treatment of EC.
